# Structure-guided computational design and mechanistic understanding of the p95HER2-targeting NAZ-mAb antibody and its variants

**DOI:** 10.1016/j.crstbi.2026.100199

**Published:** 2026-10-13

**Authors:** Puneet Rawat, Jon Amund Kyte, Victor Greiff, Esmaeil Dorraji

**Affiliations:** a Department of Immunology, University of Oslo and Oslo University Hospital, Oslo, Norway; b Department of Cancer Immunology, Institute for Cancer Research, Oslo University Hospital, Oslo, Norway; c Department of Clinical Cancer Research, Oslo University Hospital, Oslo, Norway; d Institute of Clinical Medicine, University of Oslo, Norway; e Immunoquest Therapeutics AS, Oslo, Norway

## Abstract

Human epidermal growth factor receptor 2 (HER2) is an oncogenic receptor tyrosine kinase in breast cancer and other malignancies. A subset of HER2-positive tumours expresses 611-CTF-p95HER2, a tumour-specific, hyperactive truncated isoform associated with metastasis and treatment resistance that lacks most of the extracellular domain targeted by conventional HER2-directed antibodies. We previously developed NAZ-mAb (formerly known as Oslo-2), a monoclonal antibody against 611-CTF-p95HER2. Here, we describe a computational antibody-engineering workflow for designing variants of NAZ-mAb. Starting from the sequence alone, we modelled the NAZ-mAb–611-CTF-p95HER2 complex, generated a combinatorial mutational landscape using FoldX 5.0, and prioritized candidate variants using predicted interaction energy and developability criteria. Two variants representing distinct design strategies were selected for validation: an aromatic double mutant, NAZ-mAb v1 (L:S31W/L:H107W), and a conservative single mutant, NAZ-mAb v2 (L:S31M). Both variants were successfully expressed as recombinant IgGs; NAZ-mAb v2 achieved a five-fold higher recombinant expression yield than parental NAZ-mAb, while both variants retained antigen binding with a higher apparent signal than the parental antibody in indirect ELISA. However, Biacore two-state kinetic analysis revealed weaker affinities than the parental antibody (K_D_ NAZ-mAb v1: 326.1 nM, NAZ-mAb v2: 94.5 nM vs parental NAZ-mAb: 53.3 nM). These findings show that the computational workflow can generate antigen-engaging NAZ-mAb variants, while also highlighting the limitations of fixed-backbone interaction-energy ranking as a predictor of binding affinity and yield. This study provides a practical framework and a cautionary benchmark for computationally driven, developability-aware antibody engineering in the absence of experimental structural data.

## Highlights

• Selective p95HER2 targeting may help overcome HER2 therapy resistance.
• Computational sequence-to-structure workflow designs NAZ-mAb variants for p95HER2.
• Testing variant design strategies exposed aromatic biases in energy-based models.
• Conservative point mutation boosts yield five-fold while retaining 611-CTF binding.
• Higher-order and aromatic mutations reveal limits of fixed-backbone affinity scoring.

## Graphical abstract

**Figure:**
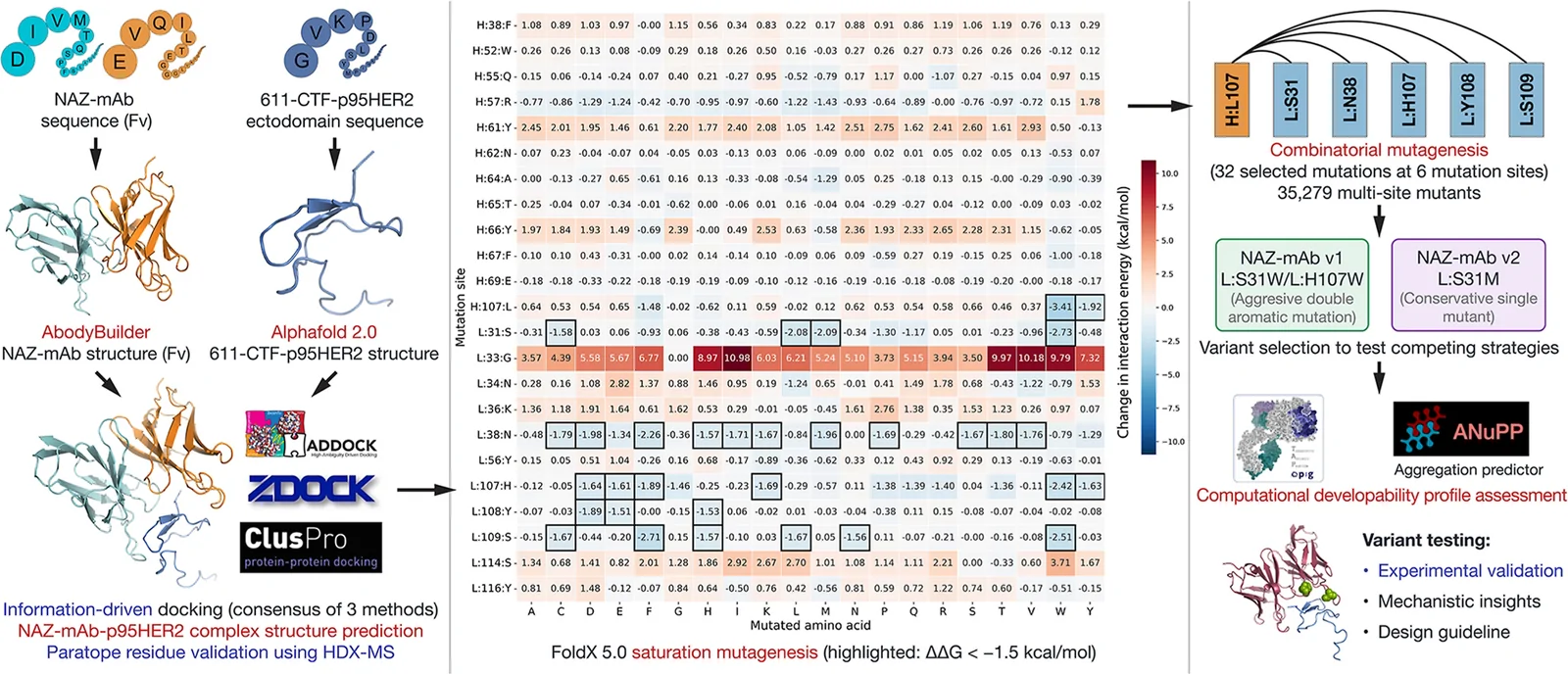

## 1 Introduction

Breast cancer is the most frequently diagnosed cancer in women, with an estimated 2.3 million new cases and 670,000 deaths globally in 2022 (Kim et al., 2025), and it accounts for nearly one-third of new cancers in women in the United States (Siegel et al., 2026). It is a highly heterogeneous disease encompassing biologically and clinically distinct subtypes with differing molecular drivers, prognosis, and treatment responses (Caswell-Jin et al., 2021). Human epidermal growth factor receptor 2 (HER2) is amplified in roughly one-fifth of breast cancers and is the target of trastuzumab and related biologics (Owens et al., 2004). HER2 carboxy-terminal fragments (CTFs), collectively termed p95HER2, can arise through more than one mechanism. Critically, the hyperactive 611-CTF isoform is generated by alternative translation initiation at the internal AUG encoding methionine 611, rather than by proteolytic shedding of full-length HER2 (Anido et al., 2006). 611-CTF retains the transmembrane and intracellular kinase domains but lacks most of the HER2 extracellular domain, creating a constitutively active receptor that promotes mammary tumour growth and metastasis in experimental models (Pedersen et al., 2009). Clinically, a high p95HER2/HER2 ratio has been associated with inferior progression-free and overall survival in trastuzumab-treated HER2-positive metastatic breast cancer (Chumsri et al., 2018; Sperinde et al., 2010). Because 611-CTF lacks the extracellular regions recognised by trastuzumab and pertuzumab, the truncated receptor is not directly engaged by these conventional HER2-directed antibodies (Scaltriti et al., 2007). Importantly, alternative translation initiation at M611 creates a distinct N-terminal extracellular context that can be selectively targeted. Although the underlying amino-acid sequence is present within full-length HER2, the N-terminal epitope exposed in 611-CTF is not accessible in the intact receptor. We previously developed NAZ-mAb, which selectively recognises 611-CTF-p95HER2 over full-length HER2 and maps to the N-terminal epitope PIWKFPDEE (Dorraji et al., 2022). NAZ-mAb therefore provides a molecularly selective targeting scaffold for the 611-CTF-p95HER2 isoform and is the engineering substrate for the present study.

Mechanistic understanding of binding behaviour, and rational improvement of an antibody, requires a structural model of the paratope–epitope interface, yet experimental structures of antibody–antigen complexes remain scarce due to technical difficulty and high cost. Recent advances across computational structural biology now make it feasible to address this gap through hybrid computational-experimental approaches at reduced cost and time. Sequence-based structure prediction yields high-quality models of both antibody (Leem et al., 2016; Ruffolo et al., 2023; Kenlay et al., 2024) and antigens (Jumper et al., 2021; Krishna et al., 2024), but prediction of the antibody–antigen complex itself lags behind (Smorodina et al., 2026). A recent benchmark study reported that more than half of the antibody–antigen complex predictions failed to reach acceptable accuracy (Xu et al., 2025), largely because the hypervariable CDR-H3 loop and the absence of co-evolutionary signal at the interface are poorly captured.

Prior knowledge of the epitope can substantially improve docking, and such information can be obtained from comparatively inexpensive experimental approaches (site-directed mutagenesis, overlapping-peptide scanning or hydrogen–deuterium-exchange mass spectrometry) rather than from structure-determination techniques (such as X-ray crystallography or cryo-EM). This restraint-guided, consensus modelling can potentially improve the antibody–antigen complex structure prediction for downstream analysis. A therapeutic antibody must satisfy two objectives simultaneously: high, specific affinity and good developability (stability, low aggregation and manufacturability). These objectives frequently conflict and must be balanced when designing new variants (Raybould et al., 2024; Makowski et al., 2022). Although many optimisation methods exist, current machine-learning approaches are constrained by limited training-set size and poor interpretability (Akbar et al., 2022). Sequence-based protein and antibody language models (e.g., ESM, AbLang, AntiBERTy) have shown strong performance for tasks such as sequence completion, humanness assessment and variant-effect prediction (Rives et al., 2021; Olsen et al., 2022; Ruffolo et al., 2021). However, these models are typically trained to capture sequence “naturalness” or require task-specific, affinity-labelled data; they do not directly report interaction energy at a defined structural interface, and they remain limited in interpretability. Moreover, many machine-learning affinity predictors have been shown to largely recapitulate stability rather than binding (de Kanter et al., 2026). We therefore deliberately used interpretable, physics-based methods for affinity and developability assessment, so that the basis of each design decision can be understood mechanistically, and validated against recombinant expression, indirect ELISA and surface-plasmon-resonance measurements. The central value of the study is mechanistic: aggressive mutagenesis of the parental antibody produced larger and less predictable changes, whereas conservative substitutions yielded only incremental effects, and the most robust strategy proved to be conservative substitution at positions not directly involved in antigen contact. We further show that empirical, fixed-backbone energy functions are systematically biased toward aromatic substitutions, which are over-rewarded through favourable packing and π-contacts despite their conformational and desolvation costs. These insights are transferable to other antibody–antigen systems where experimental structural information is lacking.

## 2 Materials and methods

### 2.1 Complex modelling and interface definition

The extracellular domain of 611-CTF-p95HER2 was modelled using AlphaFold 2.0 (Jumper et al., 2021) and the NAZ-mAb antibody scFv (and variant) structure with AbodyBuilder (Leem et al., 2016; Abanades et al., 2023). Three docking algorithms, Zdock 3.0.2 (Pierce et al., 2014), Haddock 2.4 (Honorato et al., 2024), and ClusPro 2.0 (Brenke et al., 2012) were used in information-driven docking to predict the top 10 docking poses. The experimentally-validated “MPIWKFPDEE” region in the extracellular domain of p95HER2 was provided as a potential epitope region for docking (Dorraji et al., 2022). The final complex was selected by consensus across the three methods, scored by the percentage epitope overlap.

### 2.2 Mutational scanning and combinatorial modelling

FoldX 5.0 (Delgado et al., 2019) was used to generate the mutant structures and to compute interaction energy and stability. The wild-type complex was first relaxed with the “RepairPDB” command. Paratope and epitope residues were defined by an inter-chain heavy-atom contact distance cut-off of 4.5 Å. Complete single-point saturation mutagenesis (19 substitutions per position) was performed across all paratope residues with “BuildModel” command. The interaction energy was calculated with the “AnalyseComplex” command for wild-type and each mutant structure. The change in interaction energy (Δ Interaction energy) with respect to wild-type was calculated as:

$$\Delta \;Interaction\;energy={Interaction\;energy}_{mutant}-{Interaction\;energy}_{wild-type}$$

Where, a negative change in interaction energy indicates improved binding affinity. Positions at which more than half of the 19 substitutions improved interaction energy were defined as binding hotspots. To limit the combinatorial multi-site mutation space, only high-confidence binding-improving substitutions with ΔΔG_interaction < −1.5 kcal/mol were retained. This is a pragmatic prioritisation cut-off, chosen to prioritise the strongest predicted improvements and reduce the combinatorial search space (Sapozhnikov et al., 2023; Vliora et al., 2026). An exhaustive combination of up to six substitutions produced 35,279 unique multi-site mutants, each scored for change in interaction energy relative to wild-type. All positions or mutations are defined as [chain]:[wild-type][position][mutation]. The stability of the complex was calculated using the “Stability” command in FoldX 5.0 and using DynaMut2 server (Rodrigues et al., 2021). The interaction energy was also validated using MutaBind2 server (Zhang et al., 2020). The parental and variant Fv models generated with AbodyBuilder2 were superposed and visualised in PyMOL 3.1 (https://www.pymol.org/). To quantify backbone differences, models were superposed on Cα atoms, and per-region Cα RMSD and per-residue Cα displacements were computed relative to parental NAZ-mAb.

### 2.3 Developability and aggregation profiling

Developability of selected mutants was assessed with the Therapeutic Antibody Profiler (TAP) (Raybould et al., 2024), reporting total CDR length, CDR-vicinity patches of surface hydrophobicity (PSH), positive and negative charge patches (PPC/PNC), and the structural Fv charge symmetry parameter (SFvCSP), each interpreted against the published amber/red guideline thresholds (as of June 2026). Aggregation-prone regions (APRs) were predicted with ANuPP (Prabakaran et al., 2021) using the default model threshold.

### 2.4 Gene synthesis, expression and purification

Variable heavy and light domains for the selected NAZ-mAb antibody variant were codon-optimised for human-cell expression, synthesised with cloning restriction sites, and inserted into mouse-IgG1 heavy- and light-chain expression vectors. Sequence identity was further verified by Sanger sequencing. HEK293 cells were passaged to an appropriate stage for transient transfection. Heavy-chain and light-chain expression vectors were co-transfected, and cells were cultured for approximately 6 days before harvest. Cultures were clarified by centrifugation and filtration. Antibodies were purified by Protein A affinity chromatography, eluted using citrate buffer at pH 3.0, neutralized with Tris buffer, and buffer-exchanged into PBS, and quantified by UV spectroscopy. The mammalian signal peptide MPLLLLLPLLWAGALA was used for both chains.

### 2.5 Quality control

Purified antibodies were analyzed by SDS-PAGE under reducing and non-reducing conditions. Purity and aggregation profile were assessed by SEC-HPLC and endotoxin level was measured by LAL chromogenic assay. Concentration, production yield, formulation buffer and physical inspection results were also reported.

### 2.6 Indirect ELISA binding assay

Microtitre plates were coated with the p95HER2-derived peptide antigen (MPIWKFPDEEGACQPCPINCTHSCVDLDDKGCPAEQRASPLTHHHHHH) at 2.5 μg/mL (50 μL/well) for 1 h with shaking at 300 rpm and blocked overnight at 4 °C with 1% casein in PBS. The peptide was supplied as an unmodified TFA salt (lot U2097FD150-1/PE4504); supplier quality-control documentation reported 98.7% RP-HPLC purity and an ESI-MS mass of 5426.4 Da, consistent with the theoretical mass of 5427.05 Da. Antibodies were titrated in technical duplicate as a 3-fold dilution series from 1.0 μg/mL to 0.46 ng/mL. The averaged absorbance value for each antibody concentration was reported. The bound antibody was detected with HRP-anti-IgG and TMB substrate for 10 min, stopped with 1 M HCl, and read at 450 nm.

### 2.7 Biacore kinetic analysis

Kinetics were measured on a Biacore T200 at 25 °C. Antibodies were covalently immobilised on a CM5 sensor chip by EDC/NHS chemistry, and the peptide antigen was flowed as analyte in multi-cycle kinetics over a ten-point, approximately two-fold concentration series from 5 nM to 2.5 μM at a flow rate of 40 μL/min, with a 90 s association and a 300 s dissociation phase. Responses were double referenced against a reference flow cell and buffer-only injections. Three independent runs were acquired for each antibody and fitted individually.

For each run, kinetic data were fitted across the full concentration series in Biacore T200 Evaluation Software v1.0 with both a 1:1 Langmuir binding model and a two-state (conformational change) model, applied to the same injection series. The two-state model was selected because it gave consistently lower χ^2^ and substantially reduced, less systematic residuals for all three antibodies. For the two-state model the overall equilibrium dissociation constant was calculated as K_D = (kd_1_/ka_1_)/(1 + ka_2_/kd_2_). A local refractive index (RI) term was fitted for each injection to account for bulk solvent contributions.

## 3 Results

### 3.1 An integrated computational pipeline translates sequence data into interaction models to guide combinatorial variant generation

Starting from sequence alone, we modelled the 611-CTF-p95HER2 (p95HER2) ectodomain and the NAZ-mAb Fv and assembled the NAZ-mAb–p95HER2 complex by information-driven docking, taking the consensus of three docking methods (Dorraji et al., 2022) (Fig. 1A). Using this complex as the wild-type reference, we defined the paratope as the antibody residues within 4.5 Å of p95HER2. These docking-derived interacting paratope residues agreed with our hydrogen-deuterium-exchange mass-spectrometry (HDX-MS) data (Dorraji et al., 2022). Therefore, using this complex as a wild type reference, we performed FoldX 5.0 (Delgado et al., 2019) saturation mutagenesis across the paratope, substituting each position with the remaining 19 amino acids and recording the change in interaction energy. The wild-type complex had interaction energy of −10.87 kcal/mol 14 out of 23 positions in the paratope region showed destabilization in more than 10 point mutations, where interaction energy of the mutant was less compared to the wild-type (Fig. 1B–Table S1). Among these, six positions (H:F38, H:W52, H:Y61, L:G33, L:K36, L:S114) had more than 17 out of 19 point mutations as destabilizing. These regions are potential binding hotspot regions, which might be essential for NAZ-mAb–p95HER2 binding.

**Fig. 1:**
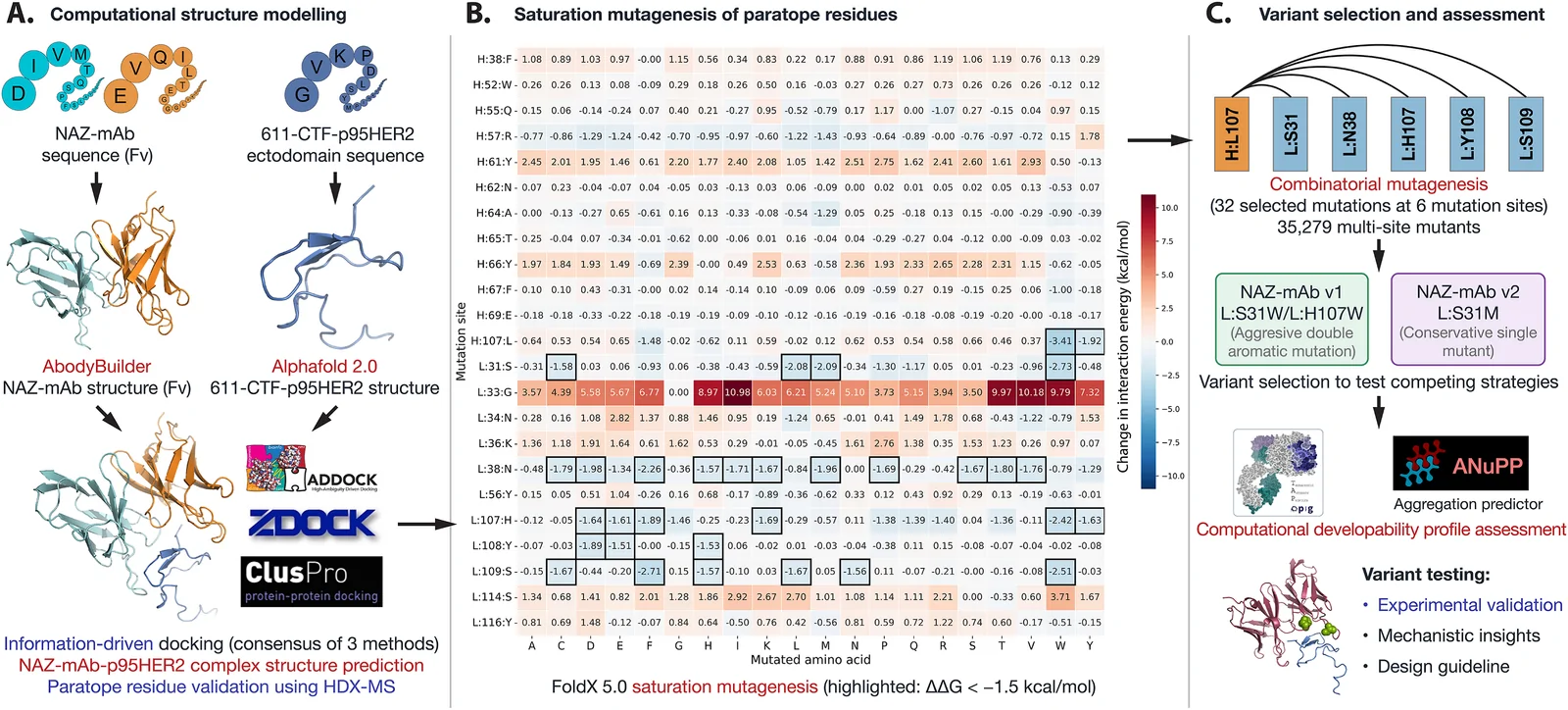
**The schematic workflow of the computational study design.** The starting points were the sequences of NAZ-mAb and 611-CTF-p95HER2 ectodomain. The computational pipeline includes modelling of the structure of both antibody and antigen, docking, mutational scanning of each paratope, combinatorial mutant generation and developability assessment. We selected two variants to test the computational variant design strategy and experimentally validated the results.

Moving from position to specific point mutation level, restricting attention to high-confidence binding-improving substitutions (Δ interaction energy < −1.5 kcal/mol) left six positions (heavy chain H:L107 and light-chain L:S31, L:N38, L:H107, L:Y108 and L:S109) and 32 qualifying point mutations. The most favourable point mutations were polar/non-polar-to-aromatic changes (Fig. 1B–Table S1), the largest being heavy-chain H:L107W (Δ interaction energy = −3.41 kcal/mol). Interestingly, the only position identified in the CDRH3 region (H:L107) had two highly stabilizing aromatic substitutions (change in interaction energy ≤ −1.5 kcal/mol). However, the other 14 mutations destabilized the NAZ-mAb–p95HER2 complex.

We further focused on the 6 paratope sites containing 32 mutations (Fig. 1C–Table 1) significantly improving binding affinity (≤−1.5 kcal/mol), mainly to accommodate the error in computational calculation and reduce the computational time to prepare all mutation combinations (23^19^ possible combinations). For all combinatorial mutations (35,279 mutations; Fig. 2), the change in interaction energy was calculated using FoldX 5.0 (Delgado et al., 2019). We observed that change in interaction energies were normally distributed (Fig. 2B), with almost all of the mutations improving the predicted interaction energy. Predicted interaction energy improved monotonically with the number of mutated sites from roughly −3 kcal/mol for the best single mutant to below −9 kcal/mol for the best six-site mutation (Fig. 2C). It may reflect the largely additive manner in which FoldX 5.0 accumulates per-site contributions and does not account for the back-bone chain flexibility and stability due to the high mutational load. The favourable interaction energy for each mutational load (no. Of site containing mutation) showed strong enrichment for tryptophan (H:L107W, L:S31W, L:H107W and L:S109W), where H:L107W was essentially obligatory in all high-ranking designs. These aromatic mutations were also high interaction energy mutants in our previous saturation mutagenesis experiment as well.

**Table 1:** The selected mutations for the combinatorial mutagenesis in up to six positions. A total of 35,279 multi-order mutations were generated. Paratope sites are denoted as chain:position:wild-type amino acid.

Paratope mutation sites	Mutation (including self mutation)
H:L107	WY
L:S31	CLMW
L:N38	CDFHIKMPSTV
L:H107	DEFKWY
L:Y108	DEH
L:S109	CFHLNW

**Fig. 2:**
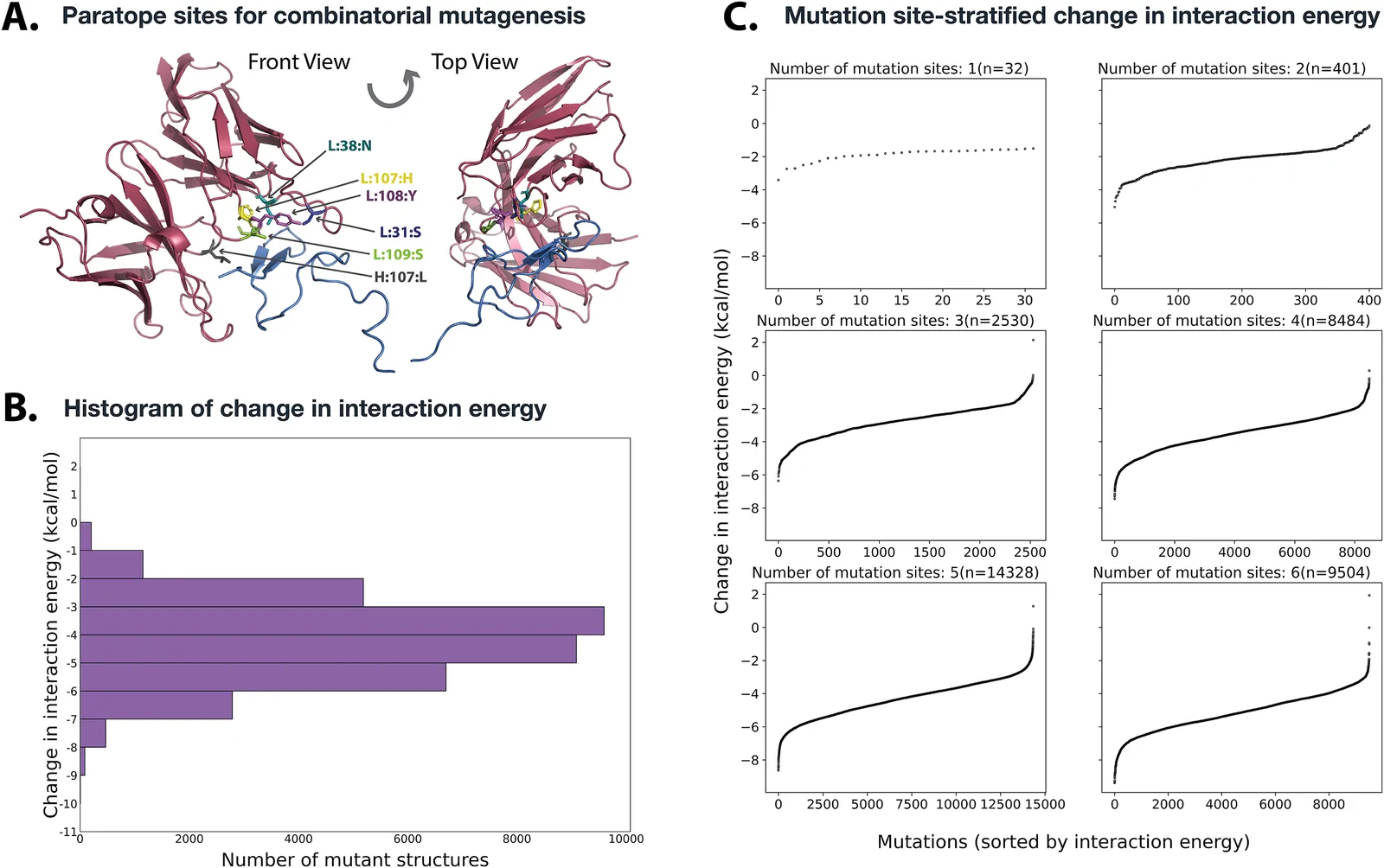
**Combinatorial mutagenesis of NAZ-mAb provides variant panels to test the design strategy. (A)** The six mutation sites, selected by saturation mutagenesis (mutating with remaining 19 amino acids) of paratope residues, are shown in the structure of the NAZ-mAb–611-CTF-p95HER2 complex as a front view (left) and top view (right). **(B)** The distribution of the change in interaction energy shown as a histogram for all combinatorial mutations, where most of the mutations show additive nature for multiple mutants and a very small percent of mutants have worse interaction energy than the wild-type. **(C)** The sorted distribution of change in interaction energy segregated by no. Of mutation sites.

### 3.2 Computational selection of NAZ-mAb variants to evaluate conservative versus aggressive rational design strategies

To provide a mechanistic understanding to design better variants, we carefully selected two variants from the 35,279 combinatorial designs for the experimental validation and hypothesis testing as follows: (i) selected an aromatic hotspot combination that tests whether combining recurrent high-impact sequentially distant aromatic substitutions improves interaction at the cost of larger physicochemical perturbation (NAZ-mAb v1; L:S31W/L:H107W), (ii) test whether a single conservative, non-aromatic light chain mutation can preserve the scaffold while modestly improving binding (NAZ-mAb v2; L:S31M). We avoided the site H:L107 in the CDRH3 region, since most mutations at this site were destabilizing except for the aromatic mutation Trp (W) and Tyr (Y) (Table S1). An additional variant bearing five light chain substitutions (L:S31W/L:N38M/L:H107W/L:Y108D/L:S109W) was also designed with significantly improved interaction energy; however, no detectable expression was obtained, likely reflecting the cumulative destabilizing effect of the mutations.

First, we computationally assessed the developability profile of the NAZ-mAb antibody variant with respect to wild-type and other clinically approved therapeutic antibodies using Therapeutic antibody profiler (TAP) (Raybould et al., 2024). It compares the developability profile of the antibody of interest with that of approved therapeutic antibodies by comparing five biophysical features: (i) Total CDR length, (ii) Patches of surface hydrophobicity (PSH), (iii) Patches of Positive Charge (PPC), (iv) Patches of Negative Charge (PNC) and (v) Structural Fv Charge Symmetry Parameter (SFvCSP) (Table S2). All parameters fell not only within the developability envelope but within the highest-density region of the latest set of 754 post-Phase-1 therapeutic Fv domains (Fig. S1, Table 2). As expected, the developability profile of NAZ-mAb v2 (L:S31M) was closest to the wild-type compared to the NAZ-mAb v1 (L:S31W/L:H107W). Additionally, we predicted aggregation-prone regions in the NAZ-mAb antibody and its variants using ANuPP (Prabakaran et al., 2021). At the default threshold 0.52, no APR was observed at this region, highlighting low aggregation propensity of the wild-type and its variants.

**Table 2:** Developability profile of NAZ-mAb antibody variants calculated from Therapeutic antibody profiler (TAP) webserver. Amber/red guideline thresholds are given in Table S2.

Metric	Wild-type	L:S31W/L:H107W	L:S31M
Total CDR Length	49	49	49
CDR Vicinity PSH Score (Kyte & Doolittle)	143.123	145.4371	142.1395
CDR Vicinity PPC Score	0.1451	0.1067	0.158
CDR Vicinity PNC Score	0.0515	0.1386	0.0531
SFvCSP Score	0	−1	−0.9

### 3.3 NAZ-mAb v2 shows a marked recombinant expression-yield advantage over parental NAZ-mAb and v1

Parental NAZ-mAb and both engineered variants were produced as mouse-IgG1 antibodies at 1 mg/mL in PBS. The variants were reported as >98% monomer by SEC-HPLC, had low endotoxin levels, and showed the expected SDS-PAGE patterns under reducing and non-reducing conditions (Table 3). Expression yield differed substantially among the three antibodies. NAZ-mAb v2 yielded 37.5 mg/L, compared with 7.5 mg/L for parental NAZ-mAb and 6.25 mg/L for NAZ-mAb v1. Thus, the single L:S31M substitution increased recombinant yield five-fold relative to parental NAZ-mAb and six-fold relative to v1, whereas v1 was produced at a yield similar to parental NAZ-mAb.

**Table 3:** Production and yield summary of the NAZ-mAb antibody variant.

Variant	Yield (mg/L)	Monomer	Endotoxin (EU/mg)	MW (Da)
NAZ-mAb	7.5	>98%	<1	146223.5
NAZ-mAb v1 (L:S31 W/L:H107W)	6.25	>98%	0.240	146,519.94
NAZ-mAb v2 (L:S31M)	37.5	>98%	0.346	146,311.74

### 3.4 Engineered NAZ-mAb variants exhibit enhanced apparent binding in ELISA but weaker monovalent affinities in Biacore analysis

In indirect ELISA both engineered variants produced higher absorbance than parental NAZ-mAb across the titration, with NAZ-mAb v2 highest and NAZ-mAb v1 matching or exceeding parental at higher concentrations (Fig. 3B). Taken alone, the NAZ-mAb v2 result would suggest improved binding.

**Fig. 3:**
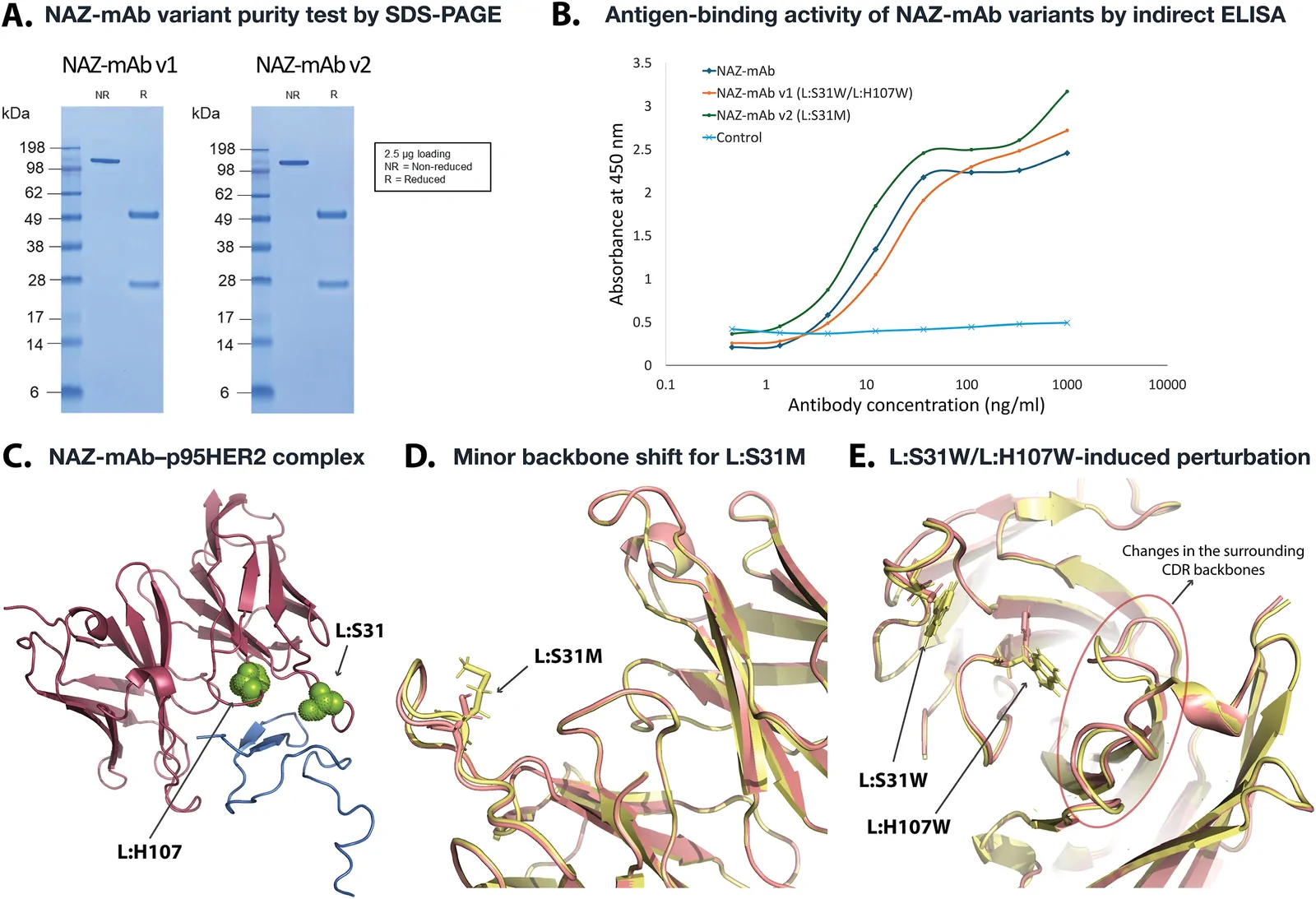
**Experimental validation and structural assessment of the variants. (A)** SDS-PAGE analysis of purified NAZ-mAb v1 and NAZ-mAb v2 under non-reduced (single band, >100 kDa) and reduced (two bands, ∼50 kDa and ∼25–28 kDa) conditions (2.5 μg loading), confirm correct assembly and purity of the expressed variants. **(B)** ELISA dose-response curves showing antigen-binding activity of NAZ-mAb (wild-type), NAZ-mAb v1, and NAZ-mAb v2 against 611-CTF-p95HER2 across a concentration range of 0.46–1000 ng/ml. Absorbance was measured at 450 nm. Both variants produced higher apparent peptide-binding signals than parental NAZ-mAb in the indirect ELISA format, with NAZ-mAb v2 generating the highest signal at the upper antibody concentrations. The control shows no binding. The FoldX 5.0 mutated structure does not account for the variation in the backbone chain upon mutations. **(C)** The structure of the wild-type NAZ-mAb (red) in complex with 611-CTF-p95HER2 ectodomain (blue), where mutation sites in the light chain are highlighted as green spheres. **(D)** The change in interaction interface upon conservative mutations L:S31M (highlighted as stick representation) leads to minor change only in CDRL1 backbone. **(E)** The change in interaction interface upon double aromatic mutations (L:S31 W/L:H107W) also shows minor variation at the CDRL1 backbone. However, these double mutations alter the structure of CDRH2 (RMSD 0.49 Å) and CDRH3 (RMSD 1.14 Å) significantly (especially at position H:L107 and H:R108). The wild-type structure is in light red color and mutated structure is in light yellow color for **(D)** and **(E)**.

However, Biacore two-state kinetics did not support a gain in affinity for either variant (Table 4). Parental NAZ-mAb had the best K_D_ at 53.3 nM, NAZ-mAb v2 was close behind at 94.5 nM, and NAZ-mAb v1 was weakest at 326.1 nM (6.1-fold loss relative to parental). The NAZ-mAb v1 deficit was driven by both a slower on-rate and a faster off-rates (Table 4). A 1:1 Langmuir model fitted to the same sensorgrams gave systematically poorer fits (Table S3, Figs. S2, S3, S4) but returned the same affinity ranking, indicating that this ordering is not an artefact of the model chosen.

**Table 4:** Biacore two-state kinetics (mean of three runs with standard deviation). The fold change is given with respect to the wild-type.

Antibody	k_a1_ (M^−1^s^−1^)	k_d1_ (s^−1^)	k_a2_ (s^−1^)	k_d2_ (s^−1^)	K_D_ (nM)	Fold change
NAZ-mAb	5.07 ± 0.13 × 10^4^	8.06 ± 0.28 × 10^−3^	3.46 ± 0.16 × 10^−3^	1.75 ± 0.22 × 10^−3^	53.3 ± 3.5	
NAZ-mAb v1	1.61 ± 0.30 × 10^4^	32.9 ± 6.98 × 10^−3^	16.2 ± 0.96 × 10^−3^	3.08 ± 0.12 × 10^−3^	326.1 ± 31.9	6.1×
NAZ-mAb v2	3.40 ± 0.06 × 10^4^	57.5 ± 2.29 × 10^−3^	12.3 ± 0.09 × 10^−3^	0.72 ± 0.05 × 10^−3^	94.5 ± 9.1	1.8×

The two assays measure different quantities. In ELISA the peptide is immobilised at high surface density and detected with bivalent IgG, so the signal reflects avidity together with detection efficiency, whereas the SPR configuration used here presents the peptide as a monovalent analyte and reports intrinsic monovalent affinity. Higher ELISA signal therefore does not establish improved intrinsic binding, and the two readouts are not in conflict (Rudnick and Adams, 2009). Critically, the experimental affinity order is the reverse of the FoldX 5.0 prediction, in which NAZ-mAb v1 (−14.27 kcal/mol) was predicted to bind more tightly than NAZ-mAb v2 (−12.96 kcal/mol).

### 3.5 State-of-the-art computational tools show inconsistent predictions for variant stability and affinity

We also used other tools to assess the binding affinity and stability of the variants with respect to the wild-type. The MutaBind2 (Zhang et al., 2020) predicted minor change in binding affinity induced by a mutation for NAZ-mAb v1 (−0.06 kcal/mol) and de-stabilization of binding for NAZ-mAb v2 (0.54 kcal/mol). The stabilization for the aromatic double mutant is aligned with the FoldX 5.0 prediction, highlighting potential biases for aromatic residues. To further assess the stability of the complex upon mutation, we estimated the change in stability upon mutations for the complex with FoldX 5.0 (Delgado et al., 2019) and DynaMut2 (Rodrigues et al., 2021). FoldX 5.0 predicted both mutations as more stable than the wild-type structure (L:S31M ΔΔG = −1.25 kcal/mol and L:S31W/L:H107W ΔΔG = −1.32 kcal/mol), aligned with the predicted affinity. This observation aligns with a previous study that showed that most affinity prediction tools are basically learning the stability prediction (de Kanter et al., 2026). On the other hand, DynaMut2 predicted stabilization for v2 L:S31M (ΔΔG = 0.18 kcal/mol) and destabilization for v1 L:S31W/L:H107W (ΔΔG = −0.39 kcal/mol). DynaMut2 uses opposite signs (positive ΔΔG is stabilizing) and only predicts the stability of the complex. The observation from different methods shows that there was no alignment in the change in binding affinity across methods. However, all tools predicted improvement in stability for the NAZ-mAb v2.

### 3.6 Bulky aromatic mutations induce significant CDR backbone rearrangements that potentially compromise binding affinity

To assess whether the FoldX 5.0-predicted affinity change reflected uncaptured backbone effects, we modelled the NAZ-mAb variants with AbodyBuilder2 and compared them with the parental model by superposing Cα atoms (Fig. 3C). The conservative variant NAZ-mAb v2 (L:S31M) was essentially superimposable on the parental (global Cα RMSD 0.11 Å; all CDRs < 0.07 Å except a 0.43 Å local adjustment at the mutated CDR-L1 site; Fig. 3D). In contrast, the light chain double aromatic mutant NAZ-mAb v1 (L:S31W/L:H107W) showed a CDR-H3 backbone rearrangement (RMSD 1.14 Å) and a smaller CDR-H2 shift (0.49 Å), with the largest per-residue displacements at H:L107 (1.91 Å) and H:R108 (1.98 Å) (Fig. 3E), despite carrying no heavy-chain mutation. As the heavy chain is identical in all constructs, this rearrangement reflects a cross-chain effect of the aromatic substitutions at the VH–VL interface consistent with the larger measured change in KD for v1.

## 4 Discussion

The entire pipeline began from sequence alone; the 611-CTF-p95HER2 ectodomain and NAZ-mAb Fv structures were modelled computationally. In the absence of an experimental co-structure, the antibody–antigen complex was assembled by information-driven docking, using the experimentally-validated “MPIWKFPDEE” region in the p95HER2 extracellular domain as the potential epitope region. This single, computationally generated complex served as the wild-type reference for all subsequent energetic calculations. The antibody–antigen interaction was dominated by CDRH2 and CDRL3 residues, consistent with prior functional evidence from HDX-MS experiments (Dorraji et al., 2022). Beyond HDX-MS, there are growing set of scalable complementary experimental strategies including deep mutational scanning and footprinting or cross-linking mass spectrometry that can independently localize paratope–epitope contacts and thereby validate docking-derived interface models (Jethva and Gross, 2023; Keen et al., 2025). FoldX 5.0 saturation scanning identified core interaction hotspots in CDRH2 that are essential for retaining binding. By contrast, substitutions in CDRL3 showed improvement in predicted binding affinity and were therefore selected as sites to engineer novel NAZ-mAb variants.

FoldX 5.0 is an empirical force field parameterised principally to predict the stability effect (ΔΔG of folding) of point mutations, computed on an essentially fixed backbone using static van der Waals packing, hydrogen bonding, and a mean-field solvation term (Delgado et al., 2019). To design novel variants and gain mechanistic insight, we first pursued an aggressive strategy by introducing two aromatic substitutions (NAZ-mAb v1, L:S31W/L:H107W), replacing a small polar residue (Ser) and a charged residue (His) with large aromatic side chains. A rigid backbone rewards new aromatic substitutions through favourable packing and π-contacts but cannot account for the corresponding costs; the conformational entropy of immobilising a bulky indole ring in the bound state, the desolvation penalty, and the backbone strain required to accommodate the larger side chains. Benchmarking of ΔΔG methods has demonstrated this failure mode, where ensemble/backbone-sampling approaches outperform fixed-backbone scoring most strongly for small-to-large side-chain mutations and for multiple simultaneous non-alanine mutations (Sanavia et al., 2020; Potapov et al., 2009; Barlow et al., 2018). Furthermore, the two aromatic mutations lie in close proximity and may sterically compete for the same interface pocket, co-distorting the loop geometry in ways that a rigid-backbone model cannot capture. As a result, the effect of multiple mutations that are likely to change the whole or part of the interface is not truly captured. Moreover, aromatic residues and proline are overestimated in the FoldX 5.0 observations (Vliora et al., 2026). This limitation is directly reflected in our data: although FoldX 5.0 predicted a substantial improvement in interaction energy for the double aromatic mutant (−14.27 kcal/mol versus −10.87 kcal/mol for wild-type), the experimentally measured K_D_ was 6.1-fold weaker than parental (326.1 nM versus 53.3 nM).

Our more conservative design NAZ-mAb v2 (L:S31M), stays inside the regime where fixed-backbone prediction is dependable and where we avoided mutation with aromatic or charged residue. The residues in the light-chain position were mutated where most substitutions seemed to improve performance, leading to the hypothesis that this position is not essential for binding. The mutated single residue was non-polar and hydrophobic (M). Although FoldX 5.0 showed a slight improvement in predicted binding affinity (dG = −12.96 kcal/mol), the measured K_D_ increased (94.5 nM), indicating slightly weaker affinity. In comparison to these mutants, the interaction energy of the wild-type was −10.87 kcal/mol and the K_D_ was 53.3 nM. This conservative single mutant nearly retained parental affinity compared to the double aromatic mutants (1.8-fold versus 6.1-fold loss). The data clearly illustrate that, for this paratope, FoldX 5.0 interaction-energy ranking is a useable hotspot-identification filter but not a quantitative affinity predictor for combinatorial designs. Although FoldX 5.0 was not able to identify the mutants that improved the K_D_, we obtained mechanistic insights into how to identify such mutants given the limitations of empirical energy-based tools.

Although the two engineered variants share the same heavy chain, their recombinant expression yields differed substantially. NAZ-mAb v2 achieved a yield of 37.5 mg/L, representing a five-fold increase over parental NAZ-mAb (7.5 mg/L) and a six-fold increase over NAZ-mAb v1 (6.25 mg/L). In contrast, v1 was produced at a yield broadly comparable to the parental antibody. These results identify v2 as the strongest production variant under the tested expression conditions, consistent with predictions from two independent stability prediction tools (FoldX 5.0 and DynaMut2). The surface-hydrophobicity score from the TAP profile decreased only marginally for v2 relative to parental NAZ-mAb (PSH 143.1 to 142.1), with both values remaining below the TAP amber threshold of 151.1. While these observations do not establish a relationship between stability or PSH and recombinant yield due to the limited dataset size, they suggest the potential for further investigation using a larger mutant dataset (Demarest et al., 2006; Knappik and Plückthun, 1995). Interestingly, we computed the FoldX folding stability (ΔΔG) for all 19 substitutions at the L:S31 position, the site of the v2 mutation (Fig. S5). L:S31M was among the most stabilizing substitutions at this position (ΔΔG = −1.33 ± 0.49 kcal/mol), whereas the other small aliphatic substitutions L:S31A, L:S31V and L:S31I were predicted to be approximately neutral (+0.02 to +0.14 kcal/mol). These predictions indicate that stability of the mutants may affect the yield. However, a systematic experimental study is needed to validate these claims. ANuPP predicted no aggregation-prone regions in parental NAZ-mAb or either engineered variant. Consistent with this, both purified variants were reported as >98% monomer by SEC-HPLC and had low endotoxin levels.

Interestingly, ELISA ranks the variants above parental, Biacore ranks them below. The different rank orders are consistent with the fact that the two assays measure binding under distinct antigen-presentation and assay geometries. Indirect ELISA uses a plate-immobilised peptide and a bivalent IgG, so its signal convolves intrinsic affinity with avidity (bivalent re-binding to densely coated antigen), coating density and detection gain, and it plateaus where avidity and antigen capacity saturate. However, the peptide also contains five cysteines and, although supplier quality-control data confirmed high purity and an intact mass consistent with the expected sequence, its cysteine oxidation state, free-thiol content and disulfide connectivity were not directly characterised. Whereas Biacore measures the monovalent K_D_. Avidity routinely masks several-fold differences in monovalent affinity, so a higher ELISA absorbance for a bivalent reagent is not evidence of tighter intrinsic binding. The mapped epitope (PIWKFPDEE) is free of cysteines, so the oxidation state is unlikely to affect the contact between the paratope and epitope directly, although it could still influence peptide presentation (e.g., oligomerization or coating density) and thus the avidity component of the ELISA signal. Biacore, with immobilised antibody and peptide analyte fitted to a two-state model, isolates the monovalent kinetic *K*_*D*_ and is the appropriate arbiter of affinity. Therefore, the variants show enhanced apparent peptide binding in the ELISA format, particularly NAZ-mAb v2, but the higher ELISA signal does not by itself establish improved intrinsic affinity.

## 5 Limitations

The most fundamental limitation is that the entire energetic analysis rests on a computationally generated, rather than experimentally determined, structure. Absolute interaction energies must therefore be treated as relative guides only, and the affinity inversion is in part attributable to compounding model error at an unverified interface. The docking-derived epitope agreed with HDX data and the hotspot map with prior functional evidence, which lends confidence to the interface, but a co-crystal or cryo-EM structure would be required to ground the energetics quantitatively. Standard MD simulations did not provide a robust basis for ranking these complexes. The antigen was highly flexible and failed to maintain its initial docked binding pose across all replicate trajectories. Consequently, reliable interface-derived metrics could not be calculated from the MD trajectories. The interaction energies reported here should therefore be interpreted as features of the static docking models.

Beyond the structural basis, only two variants out of a large design space were experimentally produced and tested, deliberately chosen to probe contrasting design strategies; the resulting conclusions are therefore hypothesis-generating and should not be over-generalised. The Biacore *K*_*D*_ values derive from a two-state fit whose micro-rate constants are model-dependent, although the overall *K*_*D*_ ranking is robust. Finally, all binding reported here is to a short synthetic peptide. Although supplier quality-control data confirmed high peptide purity and an intact mass consistent with the expected sequence, no free-thiol assay or disulfide mapping was performed; the cysteine oxidation state and disulfide connectivity therefore remain uncharacterised. Because all antibodies were compared using the same peptide lot in each assay, this uncertainty affects the absolute affinities and epitope presentation but not the relative ranking of variants, which is the basis of our conclusions. The parental NAZ-mAb was previously validated against native, membrane-embedded 611-CTF-p95HER2 by flow cytometry, immunofluorescence and immunohistochemistry, and shown to be specific for the 611-CTF isoform over full-length HER2 (Dorraji et al., 2022); whether the engineered variants retain engagement of full-length, membrane-embedded 611-CTF-p95HER2 in a cellular context remains to be established.

## 6 Conclusion

This study evaluated a sequence-to-structure computational workflow for engineering NAZ-mAb variants against the 611-CTF-p95HER2 target. As a proof-of-concept, the workflow produced two experimentally testable candidates representing contrasting design strategies: a conservative single substitution, NAZ-mAb v2 (L:S31M), and an aromatic double substitution, NAZ-mAb v1 (L:S31W/L:H107W). Although neither variant improved the measured Biacore affinity relative to parental NAZ-mAb, both retained p95HER2 engagement and showed enhanced apparent peptide binding in indirect ELISA, with NAZ-mAb v2 producing the highest ELISA signal. This improved performance in the plate-based assay does not, by itself, demonstrate improved intrinsic affinity relative to the parental antibody.

NAZ-mAb v2 emerged as the best-balanced engineered variant. It retained low-nanomolar p95HER2 binding by Biacore, showed enhanced apparent peptide binding in ELISA, and achieved a recombinant expression yield of 37.5 mg/L—five-fold higher than parental NAZ-mAb (7.5 mg/L) and six-fold higher than NAZ-mAb v1 (6.25 mg/L). This substantial yield increase is consistent with the predicted increase in stability, although the yield measurement alone cannot distinguish a stability contribution from differences in secretion or folding efficiency. NAZ-mAb v2 retained a TAP developability profile closely comparable to parental NAZ-mAb. In addition, no aggregation-prone regions were predicted for parental NAZ-mAb or either variant, and both purified variants were reported as >98% monomer by SEC-HPLC under the tested conditions.

Collectively, these findings show that fixed-backbone interaction-energy ranking alone is insufficient to predict experimentally measured kinetic affinity for higher-order mutants (Dourado and Flores, 2014). For the NAZ-mAb scaffold, the conservative L:S31M substitution provided a more productive engineering solution than stacking additional aromatic substitutions. It preserved strong target engagement, enhanced apparent binding in the ELISA format, and delivered a marked manufacturability advantage. NAZ-mAb v2 therefore represents the strongest lead from this design set for further development of p95HER2-targeting molecules.

## CRediT author statement

PR – Conceptualization, Formal analysis, Investigation, Methodology, Project administration, Software, Supervision, Validation, Writing – original draft, Writing – review and editing.

JAK – Funding acquisition, Project administration, Resources, Writing – review and editing.

VG – Resources, Supervision, Funding acquisition, Writing – review and editing.

ED – Resources, Investigation, Data curation, Validation, Conceptualization, Writing – review and editing.

## Funding

The Leona M. and Harry B. 10.13039/100007028Helmsley Charitable Trust (#2019PG-T1D011, to VG), UiO World-Leading Research Community (to VG), UiO: LifeScience 10.13039/100021494Convergence Environment Immunolingo (to VG), EU Horizon 2020 iReceptorplus (#825821) (to VG), a 10.13039/100008730Norwegian Cancer Society Grant (#215817, to VG), 10.13039/501100005416Research Council of Norway projects (#300740, #311341, #331890 to VG), a 10.13039/501100005416Research Council of Norway IKTPLUSS project (#311341, to VG). This project has received funding from the European Union's Horizon 2020 research and innovation programme under the Marie Skłodowska-Curie grant agreement No 801133 (to PR), the Norwegian 10.13039/100018696Health Region South-East (grants 2017100 and 2022070 to JAK) and the 10.13039/100008730Norwegian Cancer Society (grant 182781 to JAK).

## Declaration of competing interest

E.D. and J.A.K. are co-founders of ImmunoQuest Therapeutics AS. E.D. is affiliated with ImmunoQuest Therapeutics AS. E.D. and J.A.K. are named inventors on patent applications relating to the parental NAZ-mAb and the variants described in this study, which are exclusively licensed to ImmunoQuest Therapeutics AS. J.A.K. has received research support from Bristol Myers Squibb, F. Hoffmann-La Roche, NanoString, and NEC OncoImmunity within the past five years. V.G. declares advisory board positions in aiNET GmbH, Enpicom B.V, Absci, Omniscope, and Diagonal Therapeutics. V.G. is a consultant for Adaptyv Biosystems, Specifica Inc, Roche/Genentech, immunai, Proteinea, LabGenius, and FairJourney Biologics.
