# Supplementary material for: Structure-guided computational design and mechanistic understanding of the p95HER2-targeting NAZ-mAb antibody and its variants
Source: Curr Res Struct Biol. 2026 Oct 13;12:100199. doi: 10.1016/j.crstbi.2026.100199 (PMC13683858; doi:10.1016/j.crstbi.2026.100199)
Supplement: Supplementary file 1 [file mmc1.pdf]

Supplementary material

Structure-guided computational design and mechanistic understanding of the p95HER2-targeting NAZ-mAb antibody and its variants

Puneet Rawat [1,(0000-0002-3822-8081),*], Jon Amund Kyte [2,3,4,(0000-0002-2854-3694)], Victor Greiff [1,(0000-0003-2622-5032)], Esmaeil Dorraji [2,5,(0000-0003-1619-6610),*]

[1] Department of Immunology, University of Oslo and Oslo University Hospital, Oslo, Norway

[2] Department of Cancer Immunology, Institute for Cancer Research, Oslo University Hospital, Oslo, Norway

[3] Department of Clinical Cancer Research, Oslo University Hospital, Oslo, Norway

[4] Institute of Clinical Medicine, University of Oslo

[5] Immunoquest Therapeutics AS, Oslo, Norway

* Corresponding author

Email:

Puneet.rawat@medisin.uio.no

ED@immunoqt.com

Supplementary Figure S1. The developability profile of NAZ-mAb and its variants (green color) with respect to the 754 post-Phase-1 therapeutic Fv domains (shown as a histogram). The orange and red colors show the amber flag and red flag region, respectively, as defined by the Therapeutic antibody profiler (TAP).

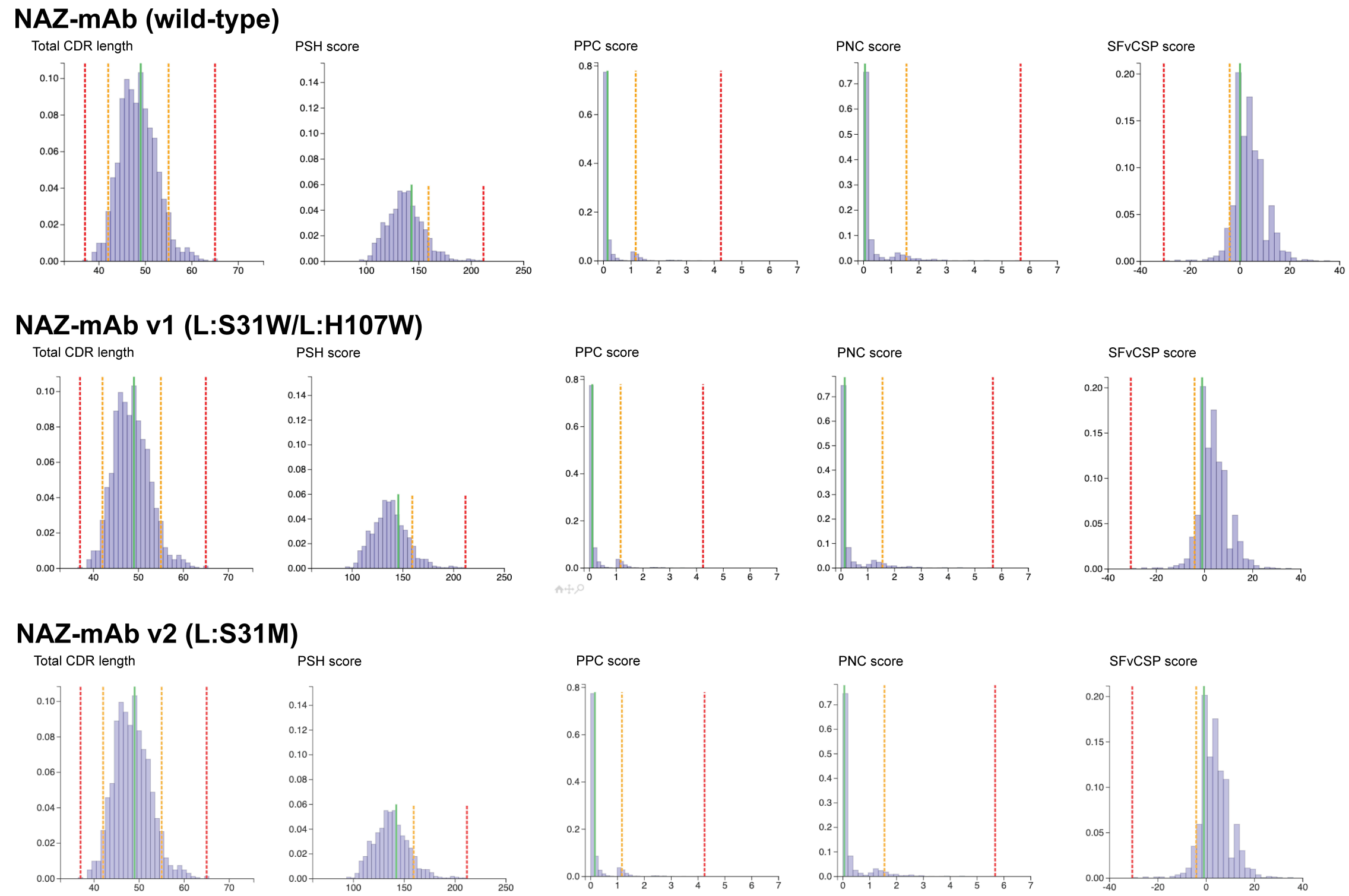

Supplementary Figure S2. Comparison of 1:1 Langmuir and two-state model fits to Biacore sensorgrams for parental NAZ-mAb. The same injection series was fitted with a 1:1 Langmuir binding model (left) and a two-state (conformational change) model (right). Coloured traces are the measured response for a ten-point, approximately two-fold peptide concentration series (5 nM to 2.5 μM); black traces are the fitted model. Lower panels show the corresponding residuals, plotted on a common scale within each row.

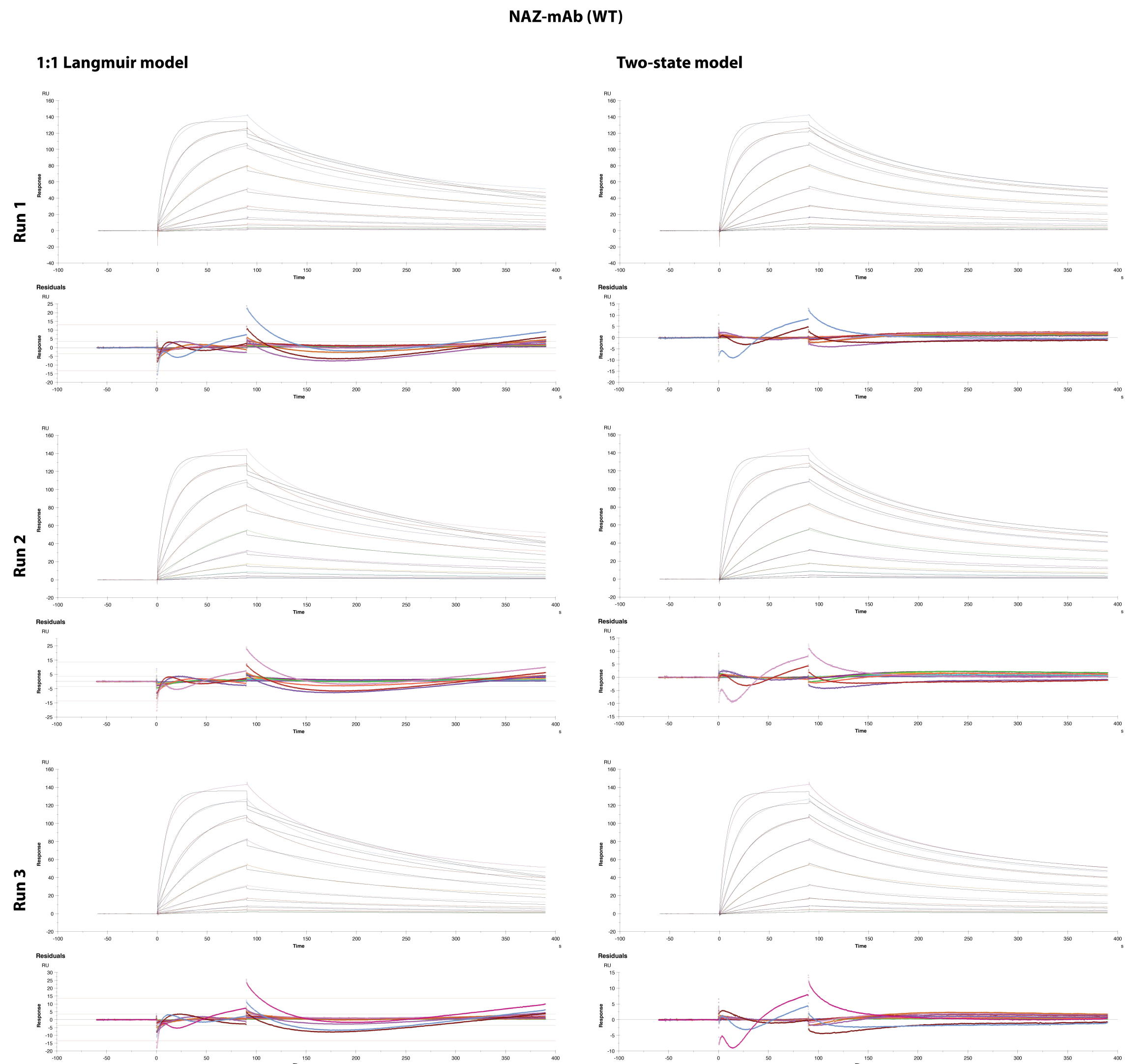

Supplementary Figure S3. Comparison of 1:1 Langmuir and two-state model fits to Biacore sensorgrams for NAZ-mAb v1 (L:S31W/L:H107W). The same injection series was fitted with a 1:1 Langmuir binding model (left) and a two-state (conformational change) model (right). Coloured traces are the measured response for a ten-point, approximately two-fold peptide concentration series (5 nM to 2.5 µM); black traces are the fitted model. Lower panels show the corresponding residuals, plotted on a common scale within each row.

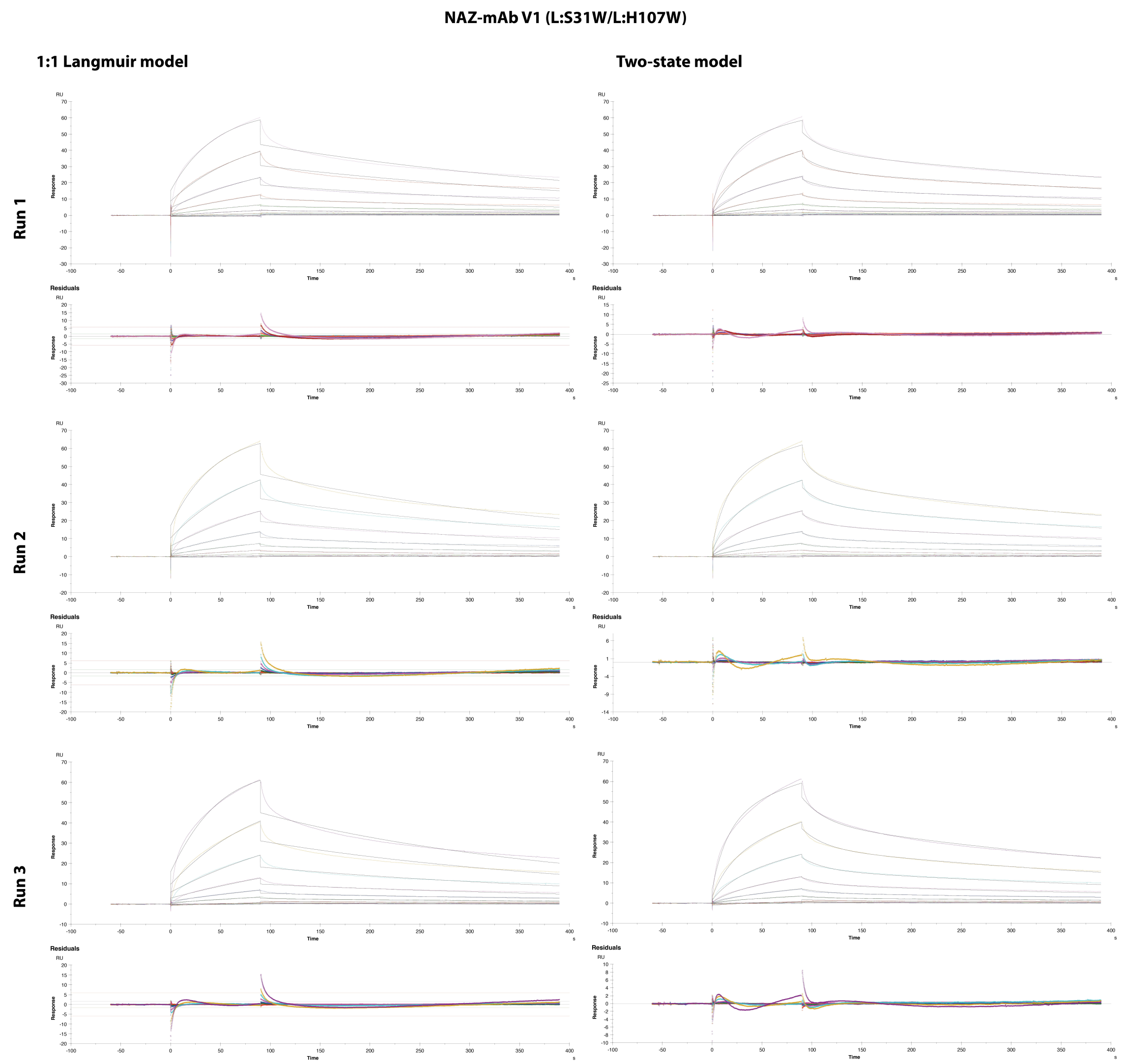

Supplementary Figure S4. Comparison of 1:1 Langmuir and two-state model fits to Biacore sensorgrams for NAZ-mAb v2 (L:S31M). The same injection series was fitted with a 1:1 Langmuir binding model (left) and a two-state (conformational change) model (right). Coloured traces are the measured response for a ten-point, approximately two-fold peptide concentration series (5 nM to 2.5 µM); black traces are the fitted model. Lower panels show the corresponding residuals, plotted on a common scale within each row.

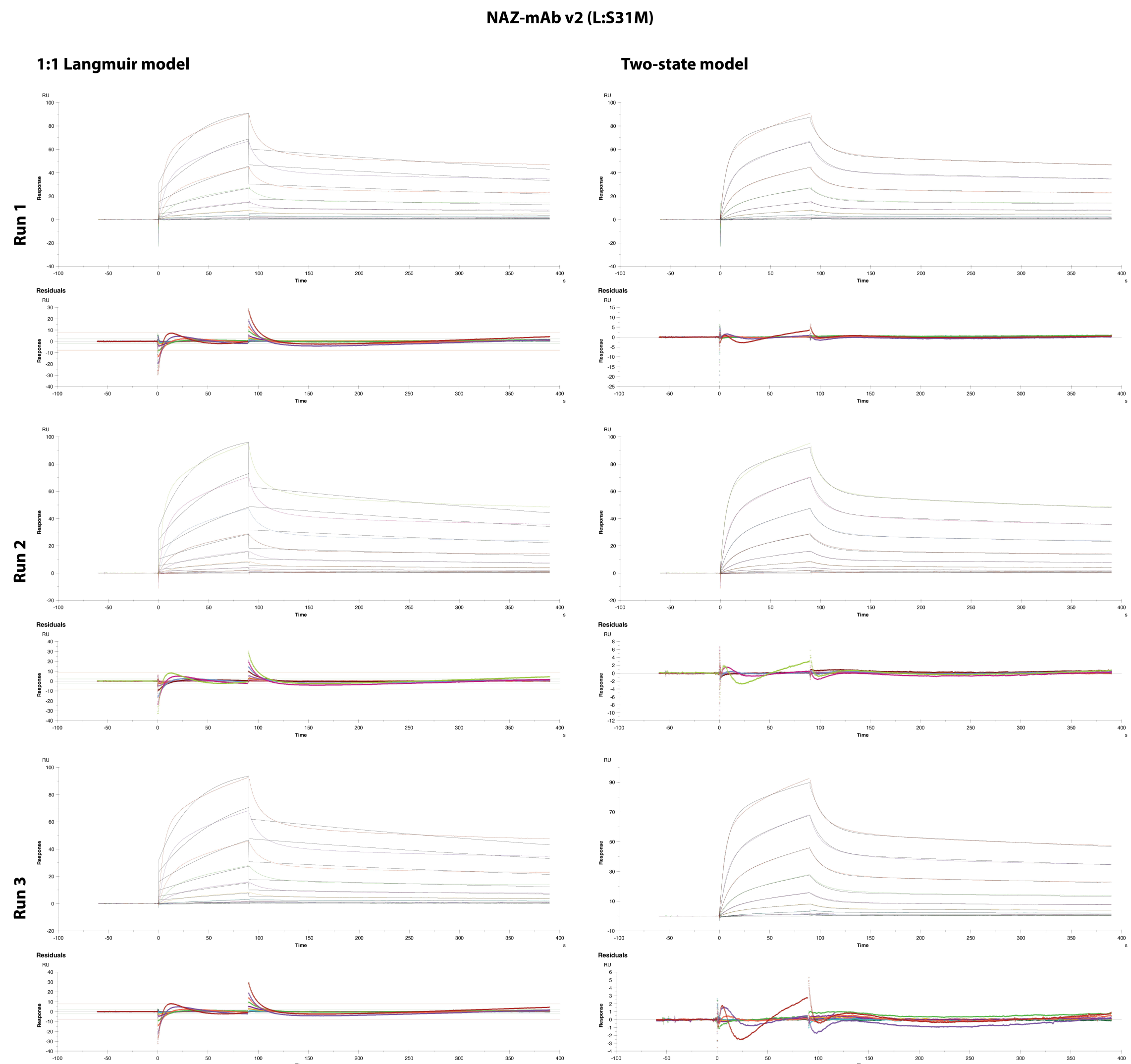

Supplementary Figure S5. FoldX-predicted folding stability for all 19 substitutions at light-chain position S31. Change in folding free energy (ΔΔG, kcal/mol) relative to the parental NAZ-mAb calculated with FoldX 5.0. Substitutions are grouped by residue class and ordered within each group by ΔΔG. Negative values indicate predicted stabilisation. Bars show the mean of five independent FoldX runs and error bars are standard deviations. The substitution S31M in NAZ-mAb v2 is outlined in bold and labelled.

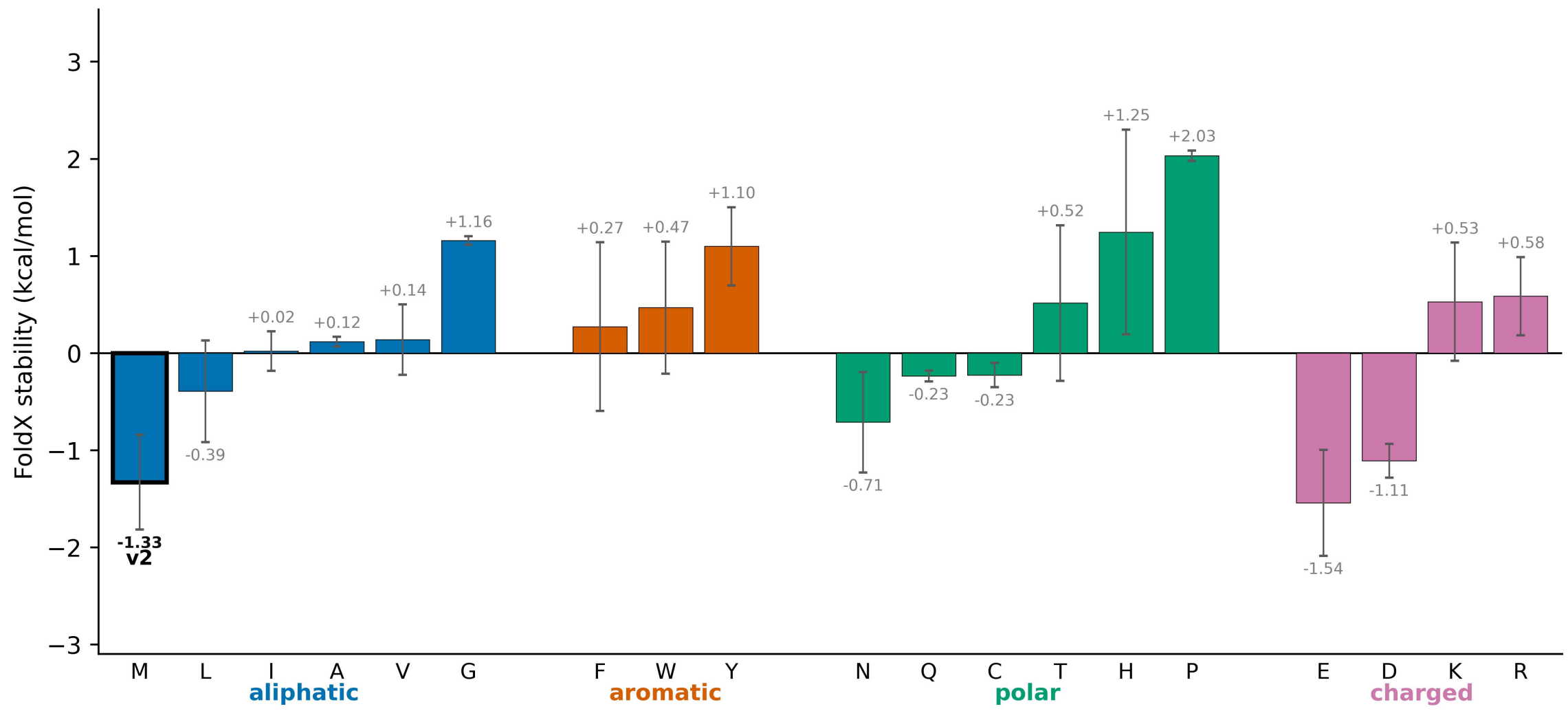

Table S1. Change in interaction energy upon point mutations for all paratope residues in NAZ-mAb antibody. Highlighted mutations have change in interaction energy less than -1.5 kcal/mol at six mutation sites. Column headers denote the mutation and row headers denote mutation sites (chain:position:wild-type amino acid). Note that antibody residue positions are given in the IMGT numbering scheme. The wild-type interaction energy was -10.87 kcal/mol.

	**A**	**C**	**D**	**E**	**F**	**G**	**H**	**I**	**K**	**L**	**M**	**N**	**P**	**Q**	**R**	**S**	**T**	**V**	**W**	**Y**
**H:38:F**	1.08	0.89	1.03	0.97		1.15	0.56	0.34	0.83	0.22	0.17	0.88	0.91	0.86	1.19	1.06	1.19	0.76	0.13	0.29
**H:52:W**	0.26	0.26	0.13	0.08	-0.09	0.29	0.18	0.26	0.5	0.16	-0.03	0.27	0.26	0.27	0.73	0.26	0.26	0.26		0.12
**H:55:Q**	0.15	0.06	-0.14	-0.24	0.07	0.4	0.21	-0.27	0.95	-0.52	-0.79	0.17	1.17		-1.07	0.27	-0.15	0.04	0.97	0.15
**H:57:R**	-0.77	-0.86	-1.29	-1.24	-0.42	-0.7	-0.95	-0.97	-0.6	-1.22	-1.43	-0.93	-0.64	-0.89		-0.76	-0.97	-0.72	0.15	1.78
**H:61:Y**	2.45	2.01	1.95	1.46	0.61	2.2	1.77	2.4	2.08	1.05	1.42	2.51	2.75	1.62	2.41	2.6	1.61	2.93	0.5	
**H:62:N**	0.07	0.23	-0.04	-0.07	0.04	-0.05	0.03	-0.13	0.03	0.06	-0.09		0.02	0.01	0.05	0.02	0.05	0.13	-0.53	0.07
**H:64:A**		-0.13	-0.27	0.65	-0.61	0.16	0.13	-0.33	-0.08	-0.54	-1.29	0.05	0.25	-0.18	0.13	0.15	0	-0.29	-0.9	-0.39
**H:65:T**	0.25	-0.04	0.07	-0.34	-0.01	-0.62	0	-0.06	0.01	0.16	-0.04	0.04	-0.29	-0.27	0.04	-0.12		-0.09	0.03	-0.02
**H:66:Y**	1.97	1.84	1.93	1.49	-0.69	2.39	0	0.49	2.53	0.63	-0.58	2.36	1.93	2.33	2.65	2.28	2.31	1.15	-0.62	
**H:67:F**	0.1	0.1	0.43	-0.31		0.02	0.14	-0.14	0.1	-0.09	0.06	0.09	-0.59	0.27	0.19	-0.15	0.25	0.06	-1	-0.18
**H:69:E**	-0.18	-0.18	-0.33		-0.18	-0.19	-0.19	-0.09	-0.1	-0.12	-0.16	-0.19	-0.16	-0.18	-0.08	-0.19	-0.2	0	-0.18	-0.18
**H:107:L**	0.64	0.53	0.54	0.65	-1.48	-0.02	-0.63	0.11	0.59		0.12	0.62	0.53	0.54	0.58	0.66	0.46	0.37	**-3.41**	**-1.92**
**L:31:S**	-0.31	**-1.58**	0.03	0.06	-0.93	0.06	-0.38	-0.43	-0.59	**-2.08**	**-2.09**	-0.34	-1.3	-1.17	0.05		-0.23	-0.96	**-2.73**	-0.48
**L:33:G**	3.57	4.39	5.58	5.67	6.77		8.97	10.98	6.03	6.21	5.24	5.1	3.73	5.15	3.94	3.5	9.97	10.18	9.79	7.32
**L:34:N**	0.28	0.16	1.08	2.82	1.37	0.88	1.46	0.95	0.19	-1.24	0.65		0.41	1.49	1.78	0.68	-0.43	-1.22	-0.79	1.53
**L:36:K**	1.36	1.18	1.91	1.64	0.61	1.62	0.53	0.29		-0.05	-0.45	1.61	2.76	1.38	0.35	1.53	1.23	0.26	0.97	0.07
**L:38:N**	-0.48	**-1.79**	**-1.98**	-1.34	**-2.26**	-0.36	**-1.57**	**-1.71**	**-1.67**	-0.84	**-1.96**		**-1.69**	-0.29	-0.42	**-1.67**	**-1.8**	**-1.76**	-0.79	-1.29
**L:56:Y**	0.15	0.05	0.51	1.04	-0.26	0.16	0.68	-0.17	-0.89	-0.36	-0.63	0.33	0.12	0.43	0.92	0.29	0.13	-0.19	-0.63	
**L:107:H**	-0.12	-0.05	**-1.64**	**-1.61**	**-1.89**	-1.46		-0.23	**-1.69**	-0.29	-0.57	0.11	-1.38	-1.39	-1.4	0.04	-1.36	-0.11	**-2.42**	**-1.63**
**L:108:Y**	-0.07	-0.03	**-1.89**	**-1.51**	0	-0.15	**-1.53**	0.06	-0.02	0.01	-0.03	-0.04	-0.38	0.11	0.15	-0.08	-0.07	-0.04	-0.02	
**L:109:S**	-0.15	**-1.67**	-0.44	-0.2	**-2.71**	0.15	**-1.57**	-0.1	0.03	**-1.67**	0.05	**-1.56**	0.11	-0.07	-0.21		-0.16	-0.08	**-2.51**	-0.03
**L:114:S**	1.34	0.68	1.41	0.82	2.01	1.28	1.86	2.92	2.67	2.7	1.01	1.08	1.14	1.11	2.21		-0.33	0.6	3.71	1.67
**L:116:Y**	0.81	0.69	1.48	-0.12	-0.07	0.84	0.64	-0.5	0.76	0.42	-0.56	0.82	0.59	0.73	1.22	0.74	0.6	-0.17	-0.51	

Table S2. The cutoffs for the developability parameters taken from the Therapeutic antibody profiler (TAP) web-server.

Developability parameters	Red flag region cutoff	Amber flag region cutoff
**Total CDR length**	<38 and 63<	<43 and 55<
**Patches of surface hydrophobicity (PSH)**	<77.6 to 182.5<	<94.9 to 151.1<
**Patches of Positive Charge (PPC)**	>3.71	>1.3
**Patches of Negative Charge (PNC)**	>3.65	>1.97
**Structural Fv Charge Symmetry Parameter (SFvCSP)**	<-19.59	<-4.95

Table S3. Goodness of fit (χ^2) for 1:1 Langmuir and two-state conformational change models fitted to identical SPR sensorgram sets for the parental NAZ-mAb and variants v1 and v2.

**Antibody**	**1:1 χ^2 values (RU^2)**	**Mean 1:1 χ^2**	**Two-state χ^2 values (RU^2)**	**Mean two-state χ^2**
NAZ-mAb	7.06, 7.43, 7.35	7.28	2.82, 2.44, 2.49	2.58
NAZ-mAb v1	0.67, 0.65, 0.58	0.63	0.28, 0.17, 0.14	0.20
NAZ-mAb v2	3.47, 3.69, 3.66	3.61	0.28, 0.16, 0.15	0.20

Note: χ^2 values are shown for three independent runs per antibody with the mean. For each antibody both models were fitted to the same injection series. All fits returned U-values below the instrument threshold for unique parameter determination, so the 1:1 model was rejected on the basis of higher χ^2 and systematically non-random residuals rather than parameter identifiability.
